# Trends in Neuropsychiatric Terminology Use Within Registered Clinical Trials for Multiple Sclerosis: A Retrospective Descriptive Analysis

**DOI:** 10.3390/healthcare13202593

**Published:** 2025-10-15

**Authors:** Braxton Phillips, Harasees Singh, Maya Morcos, Amir-Ali Golrokhian-Sani, Marc Morcos, Rui Fu

**Affiliations:** 1 Cumming School of Medicine, University of Calgary, Calgary, AB T2N 2T8, Canada; 2 Faculty of Medicine, University of Ottawa, Ottawa, ON K1H 8M5, Canada; 3 Independent Researcher, Toronto, ON M5R 0A3, Canada; 4 Departments of Community Health Sciences, Surgery & Oncology, Cumming School of Medicine, University of Calgary, Calgary, AB T2N 2T8, Canada

**Keywords:** multiple sclerosis, neuropsychiatry, bibliometric, clinical trials, industry

## Abstract

**Background/Objectives**: People with multiple sclerosis (pwMS) are known to experience more neuropsychiatric (NP) conditions compared to the general population. Clinical trials are essential for deriving effective methods to manage these conditions in this patient population, thereby optimizing their quality of life. Here, we examined the temporal trends in the inclusion of NP terms in clinical trials of multiple sclerosis (MS) to provide insights into potential gaps in research. **Methods**: Using a custom Python-based program, we analyzed the inclusion of four a priori selected NP terms (fatigue, depression, pain, and anxiety) in the description section of clinical trials of MS registered in the clinicaltrials.gov database from January 2000 to October 2024. We investigated temporal trends by correlating unique mentions of NP terms with the trial start year and examined the association of trial factors with the inclusion of any or each NP term using separate multivariable logistic regression models. We further quantified the number of trials with NP terms that explicitly examined them as a study outcome. **Results**: Of the 2674 trials, 410 (15.3%) mentioned at least one of the four NP terms. Specifically, fatigue (*n* = 293 studies), depression (*n* = 115), pain (*n* = 98), and anxiety (*n* = 49) were mentioned. Overall, the probability of trials including fatigue, depression, or anxiety, but not pain, was found to increase over time. In multivariable regression, each 1-year increase in trial start year was associated with 6% (OR 1.06, 95%CI 1.03–1.09) higher odds of including at least one NP term. Industry funding was associated with 72% lower odds (OR 0.28, 95% CI 0.20–0.39) of including any NP term. Among trials that included at least one of the four NP terms, 69.4–80.5% of them explicitly studied these terms as an outcome. **Conclusions**: Interest has increased over time in incorporating considerations on NP comorbidity in trials of pwMS. Industry-funded trials are less likely to include these considerations, which suggests a potential gap in trial design and funding resource allocation.

## 1. Introduction

Multiple sclerosis (MS) is an autoimmune disorder of the central nervous system and is characterized by inflammation, demyelination, and eventual neuronal cell death. Early neurological deficits include visual impairments, paresthesia, weakness, fatigue, spasticity, and memory impairment [1]. The etiology and pathogenesis of the disease are not well understood, though they are thought to be multifactorial and influenced by genetic and environmental factors [1]. MS is usually detected between the ages of 20 and 40, with around 10% of cases diagnosed earlier in the pediatric population [2]. Given the relatively early age of onset, the disease can cause significant psychosocial strife to individuals who live with MS and represents a profound symptom and financial burden to these individuals, their families, and society [3,4].

Compared to the general population, people with MS (pwMS), including those diagnosed in adulthood and childhood, are more likely to experience various neuropsychiatric (NP) conditions, including depression, bipolar disorder, anxiety, and psychosis [5,6,7], with the etiology most likely resulting from the interplay between disease, genetic, and psychosocial factors [8]. Interestingly, this trend is visible at least five years before diagnosis, indicating that NP conditions may be part of a ‘prodromal stage’ of the disease [9]. Indeed, depression and anxiety are the two most common comorbidities in MS [10]. Likewise, the pathophysiology of MS can cause related challenges of fatigue, pseudobulbar affect, and chronic neuropathic pain, which are often associated with worsening mental health outcomes and are often co-managed by neuropsychiatrists in clinical settings [11,12,13,14]. A Danish study previously found that the symptoms of fatigue and chronic pain were the most important correlators of poor quality of life in pwMS [15]. The uncertainty accompanying a diagnosis of MS may also predispose pwMS to greater levels of psychological distress [16]. Overall, higher levels of mental health burden in pwMS are correlated with greater productivity and employment loss [17,18].

The appropriate assessment and treatment of NP conditions is crucial to improving the quality of life and decreasing disability progression in pwMS [19,20]. Clinical trials (in particular, randomized controlled trials) contribute gold-standard evidence to clinical guidelines; however, previous studies have highlighted the dearth of clinical trials focusing on NP conditions in pwMS [7,21]. The Goldman Consensus Group and the American Academy of Neurology Guideline Development Subcommittee have called for a greater research focus to address this gap in knowledge in 2005 and 2014, respectively [19,22]. We aimed to assess the inclusion of NP terms in MS trials in the contemporary era. Specifically, the objectives of this study were to: (1) assess the temporal trends in including NP terminology in clinical trials of pwMS, as we hypothesized the inclusion to increase over time; (2) identify trial factors associated with the inclusion of NP terminology; and (3) describe the purposes of including NP terminology in these trials.

## 2. Materials and Methods

### 2.1. Study Design

This was a retrospective, descriptive, bibliometric study using publicly available trial-level data from the U.S. National Library of Medicine clinicaltrials.gov database, accessed in October 2024. No human participants were involved in this study. The authors also did not have access to information that could identify participants in the clinical trials registered in the database.

All abstracts of trials starting between 2000 and 2024 with a trial status of ‘recruiting,’ ‘active not recruiting,’ ‘terminated,’ or ‘completed’ found with the keyword search ‘multiple sclerosis’ were downloaded as .json text files. A custom Python (version 3.12.7) program, adapted from a recently published study [23], extracted the brief and detailed descriptions of each abstract and converted all text to lowercase, removing punctuation and symbols, and then split it into a list of words. Each abstract was then screened for the mention of NP terms determined a priori using the existing literature: mood disorders (‘depression,’ ‘bipolar,’ ‘mood’), ‘anxiety,’ ‘fatigue,’ chronic pain (‘pain’), pseudobulbar affect (‘pseudobulbar’), ‘distress,’ and ‘suicide.’ After a preliminary analysis of the unique abstracts featuring the above terms, we decided to retain the four terms with the most unique mentions for further analysis: “fatigue,” “depression,” “pain,” and “anxiety”. These terms encompass the two most common NP comorbidities in pwMS (depression and anxiety) and the two conditions known to adversely impact quality of life (fatigue and pain), which are additionally often co-managed by neuropsychiatrists [10,13,14,15]. In addition, many of the terms examined in this preliminary analysis only had sparse unique mentions (e.g., ‘suicide’, ‘bipolar’, and ‘psychosis’ each only had one unique mention) and were thus unsuitable for further analysis.

### 2.2. Variables

The primary outcomes were four binary variables indicating the inclusion of each NP term—fatigue, depression, pain, and anxiety—in the description section of the included clinical trials. The primary exposure was trial start year, operationalized as a continuous variable (each 1-year increase). We also extracted additional clinical trial variables from the clinicaltrials.gov database: participant age groups included, sexes included, the status of the trial as of October 2024 (active, terminated, completed), enrollment size (the number of enrolled participants), the trial funding source (industry, US government organizations, other), and trial type (observational or interventional).

### 2.3. Statistical Analysis

**Figure 1:**
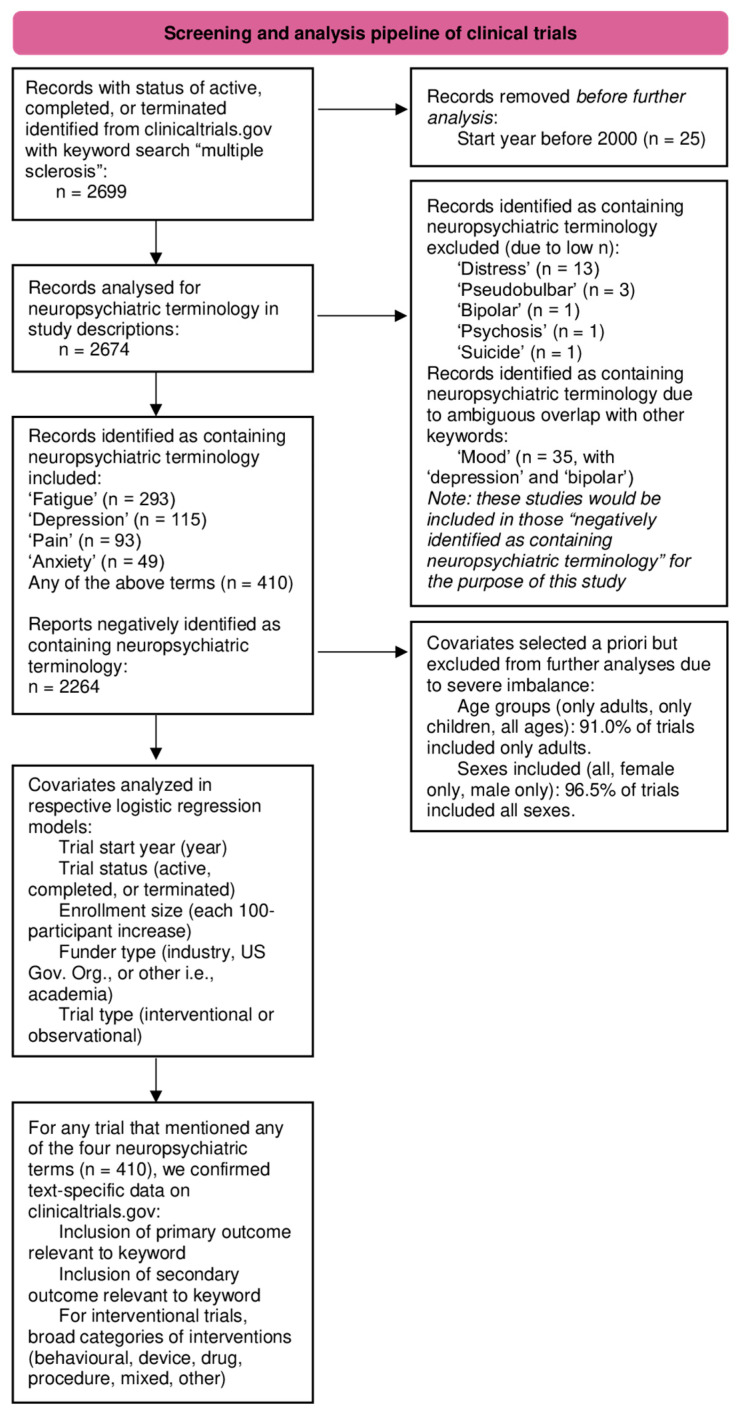
Graphical representation of the analysis plan.

Descriptive analysis was first performed on all included trials to present their characteristics, stratified by the presence of any NP terms (yes/no). Statistical comparisons of two groups were made with chi-squared tests or Fisher’s Exact Tests for categorical variables, while Mann–Whitney U tests were used to compare the median enrollment size between the two groups. For each of the four NP terms, we first used Spearman’s rank-order correlation to examine the relationship between the trial start year and the percentage of trials mentioning this term. Separate multivariable logistic regression models were then created to evaluate the association between trial start year and the inclusion of any NP term or each of the individual NP terms, adjusting for trial status, enrollment size, funding source, and trial design as independent covariates. The covariates of ages and sexes included were removed from these models as the vast majority included only adults (91.0% of the included trials) and all sexes (96.5% of the included trials), leading to an imbalance in these covariates [24]. In a secondary analysis, for trials that mentioned any of the four NP terms, we assessed whether that term was part of the outcomes being explicitly assessed in that trial or was used merely for descriptive purposes. For this analysis, two researchers manually confirmed data from clinicaltrials.gov after searching by NCT identification number to examine the characteristics of trials that screened positive for inclusion of NP terminology. To ensure that any temporal trends in NP-terminology were not inflated by trials that did not include the respective term in their trial outcomes, we repeated the correlation analysis with Spearman’s rank correlation, stratifying the temporal correlations by trials that included relevant outcomes and those that did not. We again used Spearman’s rank correlation to investigate temporal trends in intervention types studied in clinical trials with confirmed NP-relevant outcomes. All analyses were two-tailed, with statistical significance defined as *p* < 0.05. Statistical analysis was performed on R (version 4.3.3). A flowchart graphically detailing the analysis plan can be found in Figure 1.

### 2.4. Ethics Statement

This study used publicly available trial-level summary data from the clinicaltrials.gov database. No human participants were recruited for this study. As such, no formal review by a Research Ethics Board was warranted.

## 3. Results

A total of 2674 trials conducted between 2000 and 2024 were included in the study. 410 (15.3%) of the included trials mentioned at least one of the four NP terms of interest in their description. Specifically, in descending order, the following terms had unique abstract mentions: fatigue (*n* = 293, 11.0%), depression (*n* = 115, 4.3%), pain (*n* = 98, 3.7%), and anxiety (*n* = 49, 1.8%). Table 1 summarizes the characteristics of the included trials. Distributions of trial characteristics differed significantly by the inclusion status of NP terms.

**Figure 2:**
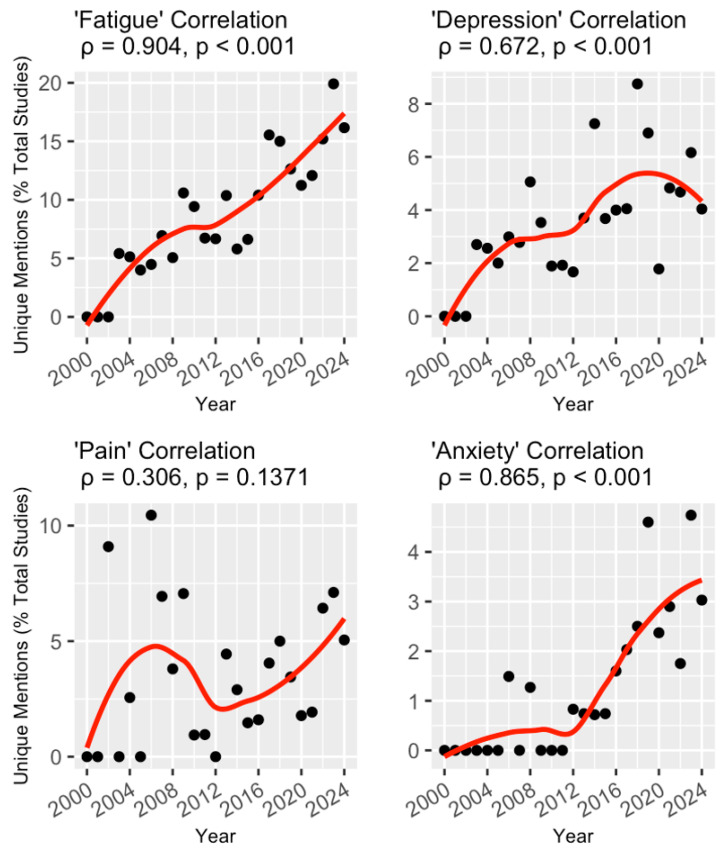
Temporal trends of trials mentioning the four NP-related keywords. For each keyword, the unique mentions (as a percentage of total studies) are plotted for each year between 2000 and 2024 (black dots) and fitted with a line of best fit using local regression (red lines). This relationship was measured with Spearman’s rank correlation, and the coefficient for each keyword is included above the corresponding graph.

Each of the four NP terms was analyzed for correlation with the start year of the trial (Figure 2). Mentions of ‘fatigue’ (ρ = 0.904, *p* < 0.001) and ‘anxiety’ (ρ = 0.865, *p* < 0.001) were positively correlated with trial start year, exhibiting seemingly linear and exponential relationships, respectively. ‘Depression’ mentions (ρ = 0.672, *p* < 0.001) were also positively correlated with trial start year, while no correlation was found for ‘pain’ mentions (ρ = 0.306, *p* = 0.14).

Multivariable logistic regression models were constructed to evaluate the association between the odds of a trial including an NP term in its abstract and its start year while adjusting for important covariates (Table 2). When examining the inclusion of at least one of the four NP terms, each 1-year increase in the trial start year (indicating more recently commenced trials) was independently associated with 6% (OR 1.06, 95% CI 1.03–1.09) increased odds. Similarly, a 1-year increase in trial start year was also associated with a 7% (95% CI 1.04–1.11), 4% (95% CI 1.00–1.09), and 17% (95% CI 1.08–1.28) higher odds of including ‘fatigue,’ ‘depression,’ and ‘anxiety’ in the abstract, respectively, while no association between trial start year and ‘pain’ was found (*p*-value = 0.48).

Furthermore, trials funded by an industry organization relative to ‘other’ funders (e.g., academia) were 79% (OR 0.21, 95% CI 0.13–0.32), 65% (OR 0.35, 95% CI 0.19–0.61), 47% (OR 0.53, 95% CI 0.29–0.90), and 88% (OR 0.12, 95% CI 0.02–0.40) less likely to include terms for ‘fatigue,’ ‘depression,’ ‘pain,’ and ‘anxiety’, respectively, as well as 72% (OR 0.28, 95% CI 0.20–0.39) less likely to include any of these NP terms.

**Figure 3:**
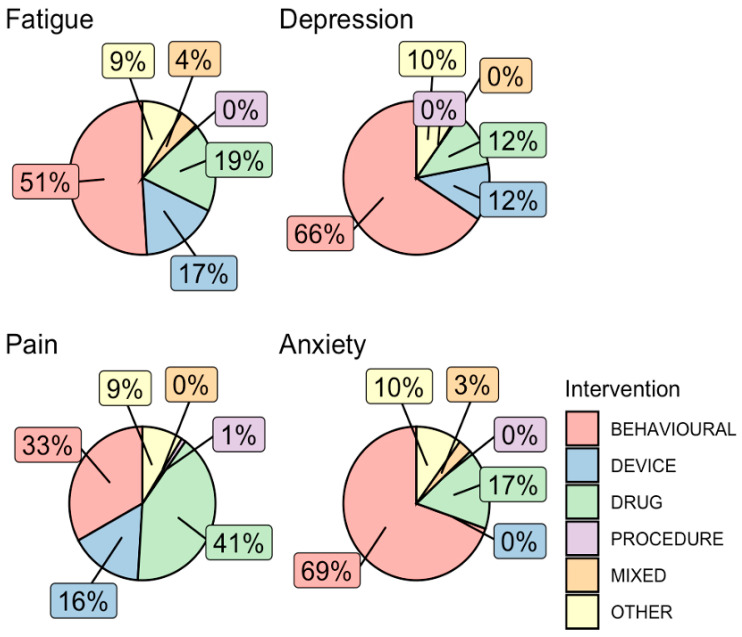
**Types of interventions in interventional trials that assessed an NP outcome.** Interventions in all trials that assessed an NP outcome were grouped into broad categories of behavioral, device, drug, procedure, mixed, or other. The percentage of trials with each intervention category was plotted in pie graphs for each NP keyword.

**Figure 4:**
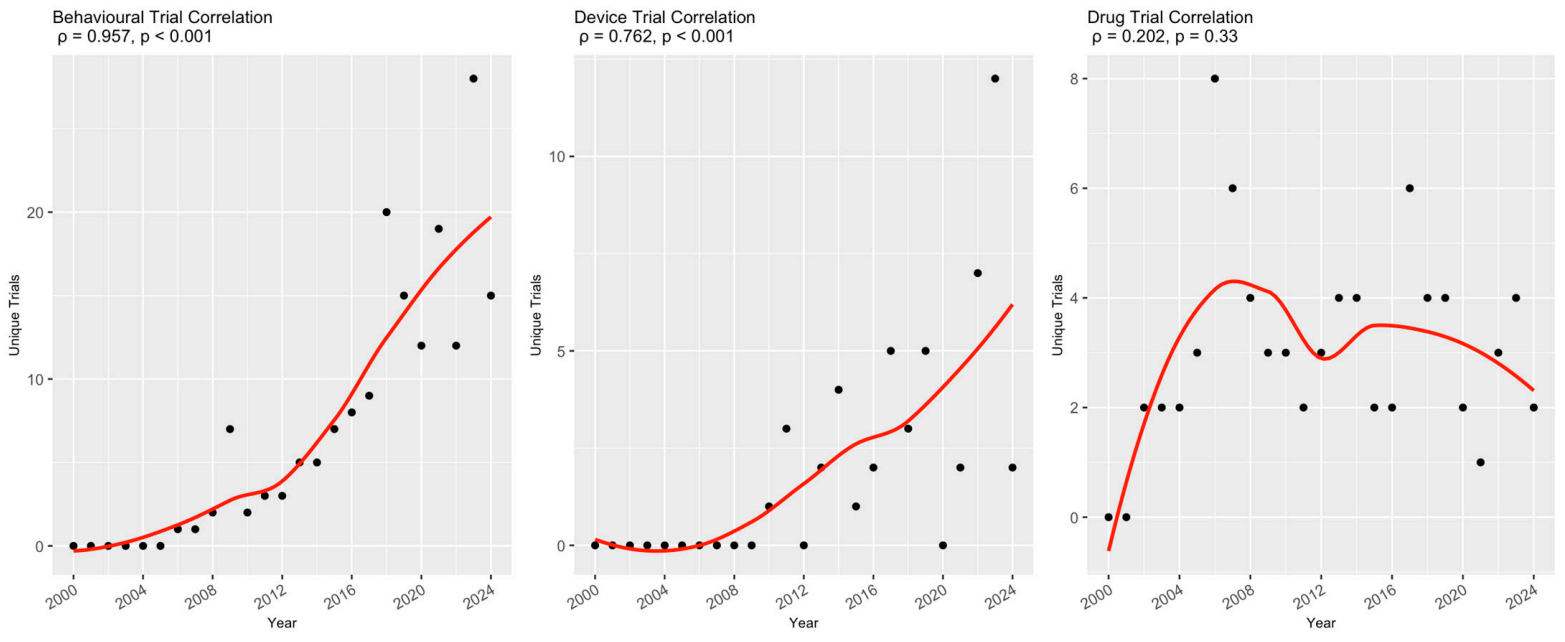
**Temporal trends of trial intervention types for trials with confirmed NP-relevant outcomes.** For each intervention type, the raw number of clinical trials is plotted for each year between 2000 and 2024 (black dots) and fitted with a line of best fit created with local regression (red lines). This relationship was measured with Spearman’s rank correlation, and the coefficient for each intervention type is included above the corresponding graph.

A secondary analysis was conducted on trials that included at least one of the four NP terms in the abstract (*n* = 410, 15.3%). Table 3 shows that the majority of them (69.4–80.5%) explicitly evaluated these terms as either a primary or secondary study outcome. These trials also had a predominantly interventional (72.5–88.3%), rather than an observational, study design. The types of intervention varied between trials for the different NP terms (Figure 3). For instance, behavioral interventions were the most common intervention type in trials that included ‘anxiety’, ‘depression’, and ‘fatigue’ as a study outcome, followed by drug- and device-based interventions, whereas for ‘pain’, the most common type of interventions were drug-based (41.0%). In an assessment of temporal trends in types of interventions studied in trials with confirmed NP-relevant outcomes, numbers of both behavioral (ρ = 0.957, *p* < 0.001) and device (ρ = 0.762, *p* < 0.001) trials were significantly correlated with increasing trial start year, while drug trials (ρ = 0.202, *p* = 0.33) were not (Figure 4).

Finally, to ensure that the temporal trends identified in NP terminology were not incorrectly inflated by trials that did not evaluate the terms as a primary or secondary outcome, trials were stratified into those that had at least one outcome measuring the relevant NP term and those that only mentioned the relevant term in the study description. The correlation analysis was repeated on these stratified groups. The unique mentions of the terms ‘fatigue’, ‘depression’, and ‘anxiety’ retained their positive and statistically significant correlations with increasing start year when only trials with relevant outcomes were considered. Interestingly, the positive correlation between unique mentions of the term ‘pain’ and the start year was significant only when considering unique mentions in trials that did not measure an associated pain outcome (Table 4).

## 4. Discussion

NP conditions are increasingly recognized as significant comorbidities in pwMS, leading to a detrimental effect on patient outcomes. To the best of our knowledge, this is the first Python-assisted, bibliometric study to comprehensively quantify the inclusion of NP terms in trials of pwMS over time. Our results are unique in the literature and may have important implications for the perceived interest among MS trialists in incorporating NP considerations and in identifying potential gaps in trial design and funding resource allocation.

Our primary finding is that the inclusion of NP terminology has significantly increased during 2000–2024 when looking at the terms ‘fatigue,’ ‘depression,’ and ‘anxiety’ in MS trials. It is important to note, however, that mention of individual NP terms is still relatively sparse across trials, ranging from a yearly peak of ~20% for ‘fatigue’ to ~5% for ‘anxiety.’ It remains to be determined whether this growing perceived research interest in NP will translate to specific interventions or guidelines for pwMS. Indeed, in our secondary analysis, trials that explicitly assessed an NP outcome were found to be mostly interventional studies focusing on behavioral interventions, with an associated temporal increase in both behavioral and device trials. Considering that behavioral trials are often employed to study interventions aimed at the treatment of mental health comorbidity, the temporal increase in these trials is consistent with the temporal increase in trials mentioning NP terminology. As an example of such interventions, the Canadian Society for Exercise Physiology has developed 24 h movement guidelines for pwMS that specifically recognize the benefits of exercise on depression and fatigue [25]. Conversely, our secondary analysis revealed that for interventional trials studying depression or fatigue as a study outcome, they were increasingly likely to focus on a device-based intervention (e.g., transcranial direct current stimulation, repetitive transcranial magnetic stimulation, or robot-assisted gait therapy); the reasons behind this finding warrant further investigations [26]. In addition, as a preliminary analysis, we grouped interventions into broadly defined categories as defined by clinicaltrials.gov, which may obscure some more nuanced trends in the literature. Future research should aim to further delineate trends in specific interventions.

Interestingly, mention of the term ‘pain’ was not found to increase consistently over time; instead, it first peaked in the 00s decade and then reached a second peak in 2024 (Figure 1). This finding is not consistent with the literature, as a recent bibliometric analysis found that general interest in pain management research has steadily increased since 1980 [27]. Pain is broadly experienced by those with MS, including from nociceptive, neuropathic, and/or mixed mechanisms, and is often described as the most bothersome possible symptom of MS; biomedical treatments can demonstrate limited efficacy [28,29]. The discrepancy between an increasing general interest in pain management research and a stagnation of such interest in MS clinical trials is difficult to resolve, and further research in this area is warranted. We further uncovered a notable trough in mentions of the term ‘pain’ around 2012, relative to the preceding and succeeding decades. We suspect that this finding may be related to a paradigm shift in MS clinical trials at this time, away from symptom-specific trials and towards an accelerated approval of disease-modifying therapies, including fingolimod and teriflunomide [30,31].

Our study also investigated the correlation of clinical trial factors with the inclusion of NP terminology. Trials being funded by an organization in the ‘industry’ category (e.g., pharmaceutical companies) were associated with significantly decreased odds of including at least one of the four NP terms studied in their abstracts. As industry-sponsored trials tend to focus on the efficacy and safety of new treatments, our findings may indicate a potential gap in trial design for testing new disease-modifying therapies beyond their clinical effectiveness in reducing relapse rates and disability progression in pwMS. Understanding the implications of these new treatments on clinically measured and patient-reported NP outcomes warrants high-quality trial data. The inclusion of simple NP screening measures, such as the Patient Health Questionnaire-9 or the Generalized Anxiety Disorder-7, in clinical trial protocols would help understand the impact of reducing disability accumulation on psychiatric comorbidity and, if successful, could provide another measure to guide physicians and patients in therapy decision-making.

Similarly, this inclusion is important to study poor NP outcomes as an adverse event. For example, adverse neuropsychiatric outcomes were an early concern for the use of interferon betas in MS, the first class of disease-modifying therapies for relapsing-remitting MS, following the attempted suicides of five participants in a pivotal trial, all in the active interferon beta-1b treatment arm [32]. The controversy around a potential increase in incidence of depression in MS patients treated with interferon betas was not resolved until subsequent trials used validated rating scales for depression, which found no significant increase, and meta-analyses confirming these conclusions were published years after drug approval [33,34]. Despite the eventual evidence against interferons causing depression, regulatory authorities frequently published warnings of a potential link, limiting access to this treatment class for pwMS with psychiatric history. Hindsight suggests that this controversy may have been limited if the original phase III clinical trials of interferon betas in MS universally incorporated validated depression rating scales, rather than relying on physician-reported adverse outcomes to assess risk of depression and suicidality [34].

In our secondary analysis, we found that for trials that were screened as having mentioned an NP term in their description section (410/2674, 15.3%), a significant portion, but not all (69.4–80.5%), explicitly assessed these NP terms as a study outcome. There are several reasons for this observation. It is possible that some trials were still in earlier phases and more focused on safety or other efficacy outcomes. As such, they may have included the NP terms in their descriptions as an area of focus for the future or as an area for improvement. This means that while these studies may not yet have established goals to assess NP outcomes, the authors are actively considering the implications of their trial on mental health and related comorbidities, and that this consideration was taken into account while designing the trial. At other times, there were clerical errors in the clinicaltrials.gov database where the authors would mention in the detailed description the NP-related outcomes for their trial, but the database would either show no outcomes at all for the trial or only show the primary outcomes, none of which were NP-related. These appeared to be errors of technology and record-keeping rather than a failure to incorporate NP consideration within the trials. Finally, some trials that mentioned these NP terms only used them to describe the manifestations of MS, as pain, depression, anxiety, and fatigue are well-known symptoms of MS. Regardless, unique trial mentions of ‘fatigue’, ‘depression’, and ‘anxiety’ still increased overtime even when restricting the dataset to trials that also included primary or secondary outcomes relevant to the analyzed NP term (Table 4).

Our findings need to be interpreted in the context of the study design. This study was based on a dataset of trials registered in the United States, which limits the generalizability of our study to other jurisdictions and healthcare systems. Priorities, including in addressing the neuropsychiatric and/or mental health aspects of MS, may differ across nations and healthcare systems. For example, recent research has highlighted that neuropsychiatry is overall still unlikely to be incorporated into formal psychiatric training across nations, including the UK, which may be limiting formal research interest in the neuropsychiatry of MS [35]. Managing MS in other nations, including developing countries, may be associated with unique MH challenges that can arise through the social determinants of health. While important, such inquiries were not within the scope of this work and require future dedicated research to elucidate. Similarly, we were only able to examine English-language trials. Future study may consider expanding our analysis to cover non-English and non-US-based trial databases to provide a more fulsome picture of the global trend in researching NP comorbidity in pwMS.

As mentioned, we did find incidences of inconsistent trial registry reporting, including trials where NP outcomes were proposed in the trial description but not stated in the listed secondary outcomes. Such clerical and technological errors do pose a risk of introducing data noise and misclassification in retrospective studies such as ours, in addition to adverse effects on the primary reporting of trial results. Quality assurance initiatives have highlighted the need for central auditing with feedback loops, establishing reporting standards, and increasing the frequency of manual reviews [36,37], to, among other reasons, ensure clear objectives, subsequent data collection, and effective reporting of secondary outcomes, including within the field of neurology [38,39]. The discrepancies uncovered in our study emphasize the need for such measures in MS clinical trials. The American Academy of Neurology has proposed 11 quality measures as essential to the care of pwMS, including validated instruments for measuring fatigue and depression [40]—we propose that such measures should also be included within MS clinical trials.

Additionally, our primary analysis focused on four a priori selected NP terms that are both highly prevalent and lead to detrimental consequences in pwMS, but there are other NP conditions that can be examined in relation to pwMS. In our preliminary analysis, we did find other NP terms, such as suicide, psychosis, and bipolar, to be present in these trials; however, these terms were rarely mentioned (by one unique trial each), prompting us to drop considerations on these terms in our primary analysis. Our search strategy was developed to be highly specific for the desired keywords, at the potential expense of sensitivity, as we did not include potential synonyms (e.g., “anxious distress”). However, the primary metric was *unique mentions* rather than *total mentions*, and, in theory, if a clinical trial were to be legitimately concerned with anxiety in pwMS, we would expect the most precise term of “anxiety” to be used at least once. We also decided to exclude the variables of age groups included and sexes included, as the overwhelming majority of trials were specific to adults and included all sexes. Future research can adapt our methodology to comprehensively characterize the breadth of NP considerations in these trials, as well as the associations with NP terms in trials that focused on pediatric populations or specific sexes.

## 5. Conclusions

This retrospective, Python-assisted, bibliometric descriptive study of clinical trials for pwMS registered on the clinicaltrials.gov database during 2000–2024 revealed an increasing annual trend in the inclusion of specific NP terminology (fatigue, depression, and anxiety) in trial descriptions. Inclusion of the term ‘pain’ was not found to increase over time. Trials receiving industry funding were less likely to include the four NP terms compared to those sponsored by other non-US government funders (such as universities), suggesting room for improvement regarding incorporating NP considerations in the design of trials aimed at testing new disease-modifying therapies. Future research can leverage our methodology to examine trends of research interest in other fields.

## Figures and Tables

**Table 1:** Characteristics of the included trials stratified by the inclusion of any NP term.

Characteristic	No NP Term Included (*n* = 2264, 84.7%) ^a^	Any NP Term Included (*n* = 410, 15.3%)	All Trials (*n* = 2674)	*p*-Value
**Age groups**				<0.001
Only adults	2037 (90.0%)	396 (96.6%)	2433 (91.0%)	
All ages	205 (9.1%)	13 (3.2%)	218 (8.2%)	
Only children	22 (1.0%)	1 (0.2%)	23 (0.9%)	
**Sexes included**				0.81
All	2182 (96.4%)	398 (97.1%)	2580 (96.5%)	
Female only	67 (3.0%)	9 (2.2%)	76 (2.8%)	
Male only	15 (0.7%)	3 (0.7%)	18 (0.7%)	
**Start year**				<0.001
2000	3 (0.1%)	0 (0.0%)	3 (0.1%)	
2001	17 (0.8%)	0 (0.0%)	17 (0.6%)	
2002	20 (0.9%)	2 (0.5%)	22 (0.8%)	
2003	35 (1.5%)	2 (0.5%)	37 (1.4%)	
2004	35 (1.5%)	4 (1.0%)	39 (1.5%)	
2005	47 (2.1%)	3 (0.7%)	50 (1.9%)	
2006	57 (2.5%)	10 (2.4%)	67 (2.5%)	
2007	61 (2.7%)	11 (2.7%)	72 (2.7%)	
2008	69 (3.0%)	10 (2.4%)	79 (3.0%)	
2009	72 (3.2%)	13 (3.2%)	85 (3.2%)	
2010	95 (4.2%)	11 (2.7%)	106 (4.0%)	
2011	95 (4.2%)	9 (2.2%)	104 (3.9%)	
2012	110 (4.9%)	10 (2.4%)	120 (4.5%)	
2013	116 (5.1%)	19 (4.6%)	135 (5.0%)	
2014	122 (5.4%)	16 (3.9%)	138 (5.2%)	
2015	123 (5.4%)	13 (3.2%)	136 (5.1%)	
2016	108 (4.9%)	17 (4.1%)	125 (4.7%)	
2017	121 (5.3%)	27 (6.6%)	148 (5.5%)	
2018	124 (5.5%)	36 (8.8%)	160 (6.0%)	
2019	146 (6.4%)	28 (6.8%)	174 (6.5%)	
2020	146 (6.4%)	23 (5.6%)	169 (6.3%)	
2021	175 (7.7%)	32 (7.8%)	207 (7.7%)	
2022	134 (5.9%)	37 (9.0%)	171 (6.4%)	
2023	155 (6.8%)	56 (13.7%)	211 (7.9%)	
2024	78 (3.4%)	21 (5.1%)	99 (3.7%)	
**Trial status ^b^**				0.011
Active	531 (23.5%)	103 (25.1%)	634 (23.7%)	
Terminated	218 (9.6%)	20 (4.9%)	238 (8.9%)	
Completed	1515 (66.9%)	287 (70.0%)	1802 (67.4%)	
**Enrollment size**				
Median (IQR)	66 (30–185)	48 (26–100)	61 (30–166)	<0.001
**Funder type**				<0.001
Other ^c^	1332 (58.8%)	345 (84.1%)	1677 (62.7%)	
US Gov. Org.	89 (3.9%)	17 (4.1%)	106 (4.0%)	
Industry	843 (37.2%)	48 (11.7%)	891 (33.3%)	
**Trial type**				<0.001
Observational	687 (30.3%)	71 (17.3%)	758 (28.3%)	
Interventional	1577 (69.7%)	339 (82.7%)	1916 (71.7%)	

^a^ We examined four NP terms, including depression, anxiety, pain, and fatigue. Trials that did not mention any of the four NP terms in the description section were deemed to have ‘No NP term included’. ^b^ Trial status was observed as of October 2024. ^c^ Funder type was defined following the official definition of the ClinicalTrials.gov, with ‘US National Institutes of Health (NIH)’ and ‘Other US Federal Agencies’ merged into ‘US Gov. Org.’ to facilitate the analysis. Other funders include individuals, universities, and community-based organizations. NP, neuropsychiatric; gov, government; org, organizations; IQR, interquartile range. **Bolded** terms in the characteristic column refer to the variables studied, while the subsequent non-bolded terms refer to the categories within that variable. The grey background highlights the Header row of the table.

**Table 2:** Multivariable logistic regression models assessing the odds of including NP keywords in the trial description.

	Any NP Keyword ^a^		“Fatigue”		“Depression”		“Pain”		“Anxiety”	
Variable	OR (95% CI)	*p*-Value	OR (95% CI)	*p*-Value	OR (95% CI)	*p*-Value	OR (95% CI)	*p*-Value	OR (95% CI)	*p*-Value
Trial start year, each 1y increase	1.06 (1.03–1.09)	<0.001	1.07 (1.04–1.11)	<0.001	1.04 (1.00–1.09)	0.046	1.02 (0.97–1.06)	0.48	1.17 (1.08–1.28)	<0.001
Trial status ^b^, vs. active										
Terminated	0.78 (0.44–1.32)	0.37	0.66 (0.32–1.24)	0.22	0.79 (0.25–2.02)	0.64	0.71 (0.25–1.79)	0.5	0.47 (0.03–2.51)	0.48
Completed	1.48 (1.10–2.01)	0.01	1.35 (0.97–1.90)	0.08	1.58 (0.94–2.70)	0.09	1.11 (0.63–1.99)	0.72	1.46 (0.72–2.99)	0.29
Enrollment size, each 100-person increase	0.95 (0.89–0.99)	0.03	0.92 (0.85–0.98)	0.04	0.96 (0.87–1.00)	0.28	0.94 (0.82–0.99)	0.24	0.99 (0.98–1.00)	0.83
Funder type ^c^ vs. ‘other’										
Industry	0.28 (0.20–0.39)	<0.001	0.21 (0.13–0.32)	<0.001	0.35 (0.19–0.61)	<0.001	0.53 (0.29–0.90)	0.03	0.12 (0.02–0.40)	0.004
US Gov. Org.	0.91 (0.51–1.54)	0.75	0.74 (0.35–1.39)	0.38	0.76 (0.23–1.89)	0.6	1.15 (0.39–2.70)	0.77	1.17 (0.19–3.99)	0.83
Trial type, vs. observational										
Interventional	2.09 (1.58–2.79)	<0.001	1.98 (1.44–2.78)	<0.001	1.21 (0.78–1.93)	0.4	3.36 (1.80–6.98)	< 0.001	2.29 (1.11–5.36)	0.04

^a^ Separate multivariable logistic regression models were built to first assess the presence of any of the four terms (fatigue, depression, pain, anxiety) and then the presence of each term. ^b^ Trial status was observed as of October 2024. ^c^ Funder type was defined following the official definition of the ClinicalTrials.gov, with ‘US National Institutes of Health (NIH)’ and ‘Other US Federal Agencies’ merged into ‘US Gov. Org.’ to facilitate the analysis. Other funders include individuals, universities, and community-based organizations. OR, odds ratio; CI, confidence interval; y, year. The Bold text and grey background highlight the header rows.

**Table 3:** **Purposes of including NP terms in trials.** By NP term, trials are stratified by having a primary, secondary, or any outcome measuring the associated term. The percentage of trials mentioning each term that are interventional (rather than observational) is included in the final column.

NP Term	Primary Outcome	Secondary Outcome	Any Outcome	Interventional Trial Design
Fatigue	117/293 (40.0%)	156/293 (53.2%)	236/293 (80.5%)	191/236 (80.9%)
Depression	27/115 (23.5%)	60/115 (52.2%)	80/115 (69.6%)	58/80 (72.5%)
Pain	54/98 (55.1%)	57/98 (58.2%)	77/98 (78.6%)	68/77 (88.3%)
Anxiety	13/49 (26.5%)	24/49 (49.0%)	34/49 (69.4%)	29/34 (85.3%)

**Table 4:** Spearman’s rank correlations of unique mentions of NP terms, with trials stratified by the inclusion/exclusion of outcomes measuring the related NP term.

NP Term	Fatigue		Depression		Pain		Anxiety	
	Correlation	*p*-Value	Correlation	*p*-Value	Correlation	*p*-Value	Correlation	*p*-Value
Outcome—Yes	0.901	<0.001	0.679	<0.001	0.275	0.18	0.832	<0.001
Outcome—No	0.629	<0.001	0.242	0.24	0.520	0.0077	0.463	0.020
All Trials	0.904	<0.001	0.672	<0.001	0.306	0.1371	0.865	<0.001

Grey background indicates the Header row of the table.

## Data Availability

The data and Python code underlying this study will be uploaded to an online repository before publication. All raw data was obtained from clinicaltrials.gov.
